# Endoparasites of *Rattus norvegicus* With Reference to Their Zoonotic Importance in an Urban District, East Kuwait

**DOI:** 10.1155/vmi/6864944

**Published:** 2026-06-12

**Authors:** Fatemah A. M. Aryan, Laila Mohammed Azad Tahrani, Osama Mohammed El-Shafei El-Azazy

**Affiliations:** ^1^ Department of Science, College of Basic Education, Public Authority for Applied Education and Training, Kuwait City, Kuwait, paaet.edu.kw; ^2^ Veterinary Laboratories, Public Authority of Agriculture Affairs and Fish Resources, P. O. Box: 21422 Safat, Kuwait City, 13075, Kuwait

## Abstract

*Rattus norvegicus* (the brown rat) is adaptable to living in urban settlements across the globe, including Kuwait where this rodent species is widespread in human habitats. It has been known that brown rats are reservoirs of many pathogens, including parasites that can pose a threat to human and animal health. Therefore, it seems paramount to study the frequency and diversity of parasite fauna of brown rats and determine which of them have zoonotic importance in Kuwait. A total of 98 brown rats were trapped from an urban district, eastern Kuwait. Blood, feces, visceral organs, and muscles were examined grossly, microscopically, and immunologically for the detection of endoparasites. The study identified 13 species, including 3 cestodes, 4 nematodes, and 6 protozoa. *Trichosomoides crassicauda* was the most prevalent parasite (35.7%), followed by *Rodentolepis nana* (32.7%), *Hymenolepis diminuta* (23.5%), and *Taenia taeniaeformis* (23.5%). The least prevalent parasite was *Toxoplasma gondii* (1.0%), detected in only one rat. The overall prevalence of infection was 88.8% (87/98), with 33.7% positive for one parasite species, and 55.1% of infected rats had mixed infections. The infection rates were statistically insignificantly higher in young rats and females than in adults and males, respectively. The overall prevalence was significantly higher (*p* < 0.05) in the wet season than in the dry season. The current study revealed that brown rats harbored 4 parasite species that are of zoonotic importance and 3 potentially zoonotic parasites, and 7 parasite species are reported for the first time in Kuwait.

## 1. Introduction

Rodents constitute 40% of mammals on the earth, with the identification of over 2000 species in 29 families, of which family Muridae comprises small rodents, for example, rats, mice, gerbils, and jirds [1]. Apart from rodent‐devastating activities, acting as reservoirs and spreading zoonotic diseases is a major concern [2]. Rats and mice, the commensal rodents, dwelling in human habitats in urban and semi‐urban regions, have captured worldwide attention and have been intensively investigated over the last decades with respect to the parasitic infection [3, 4].

*Rattus norvegicus* (brown rat) is a cosmopolitan rodent species, adapted to living in many environments, particularly urban habitats across the globe [5]. This includes Kuwait, where the density of this pest is high in urban areas. According to the Kuwaiti Ministry of Health [6], some areas are plagued by brown rats, and infestation rates vary between 17% and 62% of the examined buildings. As urban settings represent a significant portion of the ecosystem experienced by the human population, pathogens carried by brown rats are candidates for spillover into humans. For example, brown rats can carry zoonotic pathogens, for example, hantaviruses [7] and *Leptospira* spp. [8], and are incriminated as a source of infection with murine typhus [9] in Kuwait. In Iran, the neighbor country of Kuwait, brown rats are reservoirs of many zoonotic diseases, for example, tick‐borne relapsing fever, salmonellosis, bartonellosis, leptospirosis, and Q fever [10, 11].

A few publications on parasites of rodents [9, 12], including the brown rat, appeared in Kuwait in the 1980s and 1990s. This research mainly provided data on parasite fauna in rodents but did investigate them as possible reservoirs for zoonotic parasites and discuss rigorously their implications in public health and veterinary medicine. In addition, these observations were published locally and not widely disseminated beyond government reports and local meetings.

Moreover, since the appearance of these publications, major social, economic, and demographic changes have happened in the country, with possible changes in the biodiversity and rodent/parasite community structure. Therefore, it seems paramount to alert and regularly update our knowledge about rodent parasites in the Kuwait setting, with emphasis on their threat to human and animal health.

Studying the variations in parasite species community structure among rodent host species in different geographical areas provides not only data on community diversification but also sheds light on which rodent species are most likely to serve as reservoirs of existing and possibly emerging diseases in each area [13]. In addition, the determinants (intrinsic or extrinsic) of parasite species composition and diversity have been the subject of many studies [14]. These determinants (factors) exert an important influence in shipping the component community structure in ways that vary from site to site [15]. As climatological conditions, ecological factors, and ecosystem patterns are different in various geographical areas, the research results obtained from other studies on the structure and composition of parasite communities in rat species cannot be useful to know the parasite diversity and frequency and evaluate the role of rodents as carriers of parasitic zoonosis in Kuwait.

The current study aimed to assess the endoparasite infections of brown rats and refer to their zoonotic and veterinary importance in an urban area in Kuwait. Also, we considered some extrinsic (e.g., climate and season) and intrinsic (e.g., sex, age, and density of host) factors playing a role in the prevalence and diversity of parasite species.

## 2. Materials and Methods

### 2.1. The Study Area

Jleeb Al‐Shuyoukh is an urban district (29° 16′ 0″ N 47° 56′ 0″ E), located in Farwaniya Province, Eastern Kuwait. It is densely inhabited by poorly housed migrant workers of low income. The ecological factors prevail in this residential neighborhood environment with low hygiene and accumulation of wastes, giving reason for the high densities of rat populations.

### 2.2. Rodent Trapping

**FIGURE 1:**
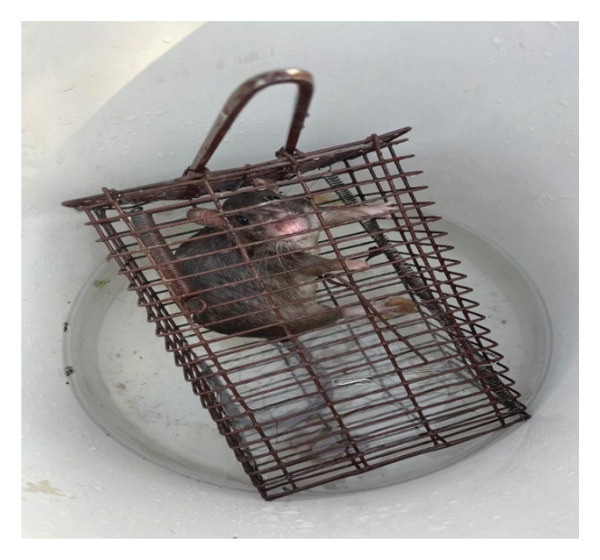
A trap for catching rats.

**FIGURE 2:**
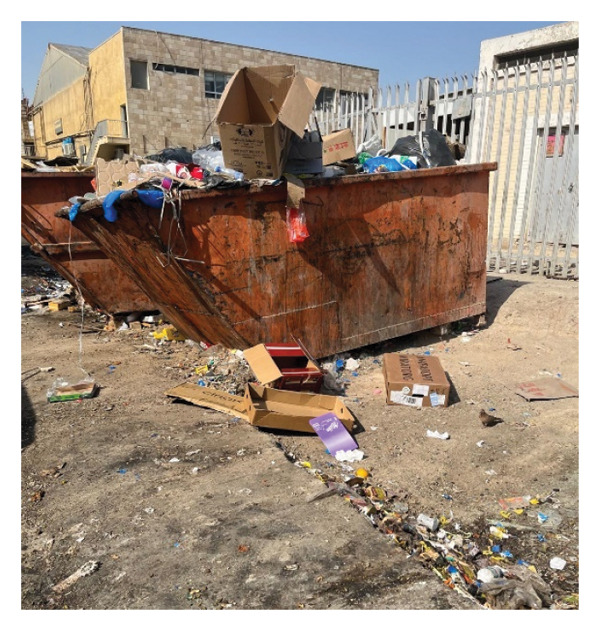
A site for sampling rats.

Between January 2023 and January 2024, rodents were captured alive using 10 special steel wire traps (38 cm L × 23 cm W × 19 cm H) (Figure 1) with slices of tomato, dried fish, pieces of cheese, or bread as bait. The traps were distributed with the help of local people in the different selected sites at homes or beside the rodent feeding resources, for example, garbage containers (Figure 2). The traps were placed in the evening and checked in the early morning. The traps, which caught rodents, were placed in plastic bags and transferred within a few hours to the laboratory for examination. Of the rat total number (*n* = 98) captured across one year, 31 (31.6%) were juveniles and 67 (68.4%) were adults, which included 32 (32.7%) males and 35 (35.7%) females. The number of rats captured in the wet season (78; 79.6%) was higher than that trapped in the dry season (20; 20.4%).

### 2.3. Rodent Examination for Parasites

In the veterinary laboratory, Public Authority of Agriculture and Fish Resources (PAAFR), Kuwait, the brown rats were anesthetized using chloroform (CHCL_3_), then identified to species level and age differentiated to adult and young (juvenile) according to [9, 15]. The rats were treated carefully without stress in adherence to the guidelines issued by the Norwegian Committee for Research Ethics in Science and Technology [16]. After anesthesia, blood was collected from the heart of each rat to be tested for *Toxoplasma gondii* and *Leishmania* spp. antibodies using immunochromatographic assays (VDRG Toxoplasma; Leishmania Ab Rapid tests, MEDIAN Diagnostics Inc., Korea). The rapid tests were performed according to the manufacturer’s instructions. Giemsa‐stained blood smears were prepared and examined under the light microscope for detection of blood parasites.

The abdomen, chest, and pelvis of each rodent were opened to remove the internal organs, which were placed in separate Petri dishes to be examined for parasites; for example, the liver for *Taenia taeniaeformis* and *Calodium hepaticum*, the lungs for *Angiostrongylus cantonensis*, and the urinary bladder for *Trichostomoides crassicauda* (bladder threadworm). For inspection of *Trichinella* spp. and *Sarcocystis* spp., small pieces of thigh muscles (1–3 mm) were compressed between 2 slides and examined under the microscope. In addition, pieces of muscles were placed in 10% formaldehyde for histopathological examination. For molecular studies to identify *Sarcocystis* spp., muscle samples from *Sarcocystis*‐infected rats were stored in 70% alcohol and shipped to the Laboratory of Molecular Ecology (Nature Research Centre, Vilnius, Lithuania).

The alimentary tract was removed and divided into the stomach, small intestine, and large intestine; each was placed in a separate Petri dish and opened. The contents were washed with normal saline, and the mucosa was scraped. The worms were collected with naked eyes or under a stereomicroscope. Nematodes were cleared in lactophenol, while cestodes were stained with alum carmine, passed in ascending grades of ethanol, cleared in xylene and clove oil, and mounted in Canada balsam [17].

Fecal samples were removed from large intestines, and fecal smears were prepared and stained with Ziehl–Neelsen (ZN) to detect *Cryptosporidium* spp. Feces were also used for the detection of *Cryptosporidium* spp. and *Giardia* spp. using immunochromatographic assay (Crypto/Giardia Duo‐Strip Diagnostic Test; CORIS BioConcept, Belgium). The rapid test was performed according to the instructions of the manufacturer. In addition, the fecal samples were examined by standard floatation methods using zinc sulfate (specific gravity 1.2) for protozoal detection [18]. The detected *Eimeria* oocysts were placed in 2.5% potassium dichromate (K_2_Cr_2_O_7_) (Ajax Chemicals, A Division of Clyde Industries Limited, Australia) for sporulation to be identified according to Levine and Ivens [19]. *E. miyairii* can be differentiated from *E. nieschulzi* by morphometric characteristics. *E. miyairii* sporulated oocysts were spherical and slightly larger, having a rough, 2‐layered, brownish, radially striated wall. While *E. nieschulzi* sporulated oocysts were elliptical with tapering ends, having a smooth, one‐layered, colorless wall.

### 2.4. Identification of Helminths

The helminths were identified based on the keys given by Khalil et al. [20] for cestodes and Anderson et al. [21] for nematodes as well as the descriptions and illustrations reported by El‐Azazy [22] and Al‐Behbehani [9]. In addition, specimens of some helminths were sent to Mike Kinsella, Helm West Laboratory, Missoula, MT, United States, and Vasyl Tkach, Department of Biology, University of North Dakota, ND, United States, for confirmation or identification of some parasites to the species level. Voucher specimens were deposited in the University of Nebraska State Museum with accession number P‐2024‐028.

### 2.5. Statistical Analysis

The comparison between the rates of parasitic infections in different brown rat age groups and genders as well as between wet and dry seasons was performed using the chi‐square test (Minitab software, State College, PA), with a 5% significance level.

## 3. Results

### 3.1. Rodent Species

During this study, 98 brown rats were captured from the study area. All rats appeared healthy without clinical signs. In addition, a few numbers (*n* = 3) of house mice (*Mus musculus*) were trapped.

### 3.2. Prevalence of Parasitic Infection in *R. norvegicus*

The necropsy of rats and their examination by different parasitological methods revealed that 87 were infected with different parasites; however, neither blood parasites nor trematodes were encountered. Also, *Leishmania* antibodies could not be detected by the immunochromatography assay.

The overall prevalence of infection was 88.8% (87/98), with 33.7% positive for one parasite species, and 55.1% of rats harbored mixed infections (Table 1).

**TABLE 1:** Parasite species load in examined *R. norvegicus*.

Rodent species	No. of parasite species	Total rodent positive (%)
*R. norvegicus*	1	33 (33.7)
	2	34 (34.7)
	3	15 (15.3)
	4	4 (4.0)
	5	1 (1.0)

The prevalence of parasitism in young rats (93.5%) was higher than that in adults (89.6%), and the infection rate was higher in males (90.6%) than in females (88.6%); however, these differences were not statistically significant (*p* > 0.05). The results showed that brown rats harbored 13 parasite species, including 3 cestodes (*Rodentolepis nana, Hymenolepis diminuta,* and *Cysticercus fasciolaris*, the metacestode of *T. taeniaeformis*), 4 nematodes (*T. crassicauda*, *Syphacia* sp.; *Streptopharagus kuntzi; Gongylonema neoplasticum*), and 6 protozoa (*Eimeria miyairii*, *E. nieschulzi*, *Sarcocystis cymruensis*, *Cryptosporidium* sp., *Giardia* sp., and *T. gondii*). Table 2 displays the infection rates of different parasites. *T. crassicauda* was the most prevalent parasite (35.7%), followed by *R. nana* (32.7%), *H. diminuta* (23.5%), and *T. taeniaeformis* (23.5%). The least prevalent parasite was *T. gondii* (1.0%) in only one rat.

**TABLE 2:** Prevalence of parasitic infections in the examined *Rattus norvegicus* according to their age and gender.

Parasite species	All rats (*n* = 98)^∗^	Adult (*n* = 67)	Young (*n* = 31)	Male (*n* = 32)	Female (*n* = 35)
	No. infected (%)	No. infected (%)	No. infected (%)	No. infected (%)	No. infected (%)
*H. diminuta*	23 (23.5)	19 (28.4)	4 (12.9)	7 (21.9)	12 (34.3)
*R. nana*	32 (32.7)	17 (25.5))	15 (48.4)	8 (25.0)	9 (25.7)
*T. taeniaeformis*	23 (23.5)	21 (31.3)	2 (6.5)	11 (34.4)	10 (28.6)
*T. crassicauda*	35 (35.7)	31 (46.3)	4 (12.9)	15 (46.9)	16 (45.7)
*Syphacia* sp.	10 (10.2)	2 (3.0)	8 (25.8)	1 (3.1)	1 (2.9)
*S. kuntzi*	4 (4.1)	4 (6.0)	—	3 (9.4)	1 (2.9)
*G. neoplasticum*	8^∗∗^ (8.2)	7 (10.4)	1 (3.2)	5 (15.6)	2 (5.7)
*Eimeria miyairii*	6 (6.1)	2 (3.0)	4 (12.9))	2 (6.3)	—
*Eimeria* *nieschulzi*	5 (5.1)	2 (3.0)	3 (9.7)	2 (6.3)	—
*S. cymruensis*	13 (13.3)	10 (14.9)	3 (9.7)	4 (15.5)	6 (17.1)
*Cryotosporidium* sp.	2 (2.0)	—	2 (6.5)	—	—
*Giardia* sp.	2 (2.0)	—	2 (6.5)	—	—
*T. gondii*	1 (1.0)	—	1 (3.2)	—	—

^∗^Number of rats examined.

^∗∗^Number rats with worms and eggs.

The prevalence of helminth infections was evaluated according to the occurrence of worms in their predilection sites. *G. neoplasticum*, which was differentiated from other species by the presence of alae and the length of spicules in males as well as the presence of few or no cuticular bosses at the anterior end, could be detected only after scratching the stomach mucosa, where they live. Two male worms were encountered in only 1 rat. Although no intact females but unidentifiable worm fragments could be observed in the gastric ingesta of other rats, *Gongylonema* spp. eggs were found in their feces. Therefore, the prevalence of this nematode was estimated according to the occurrence of worms in the gastric ingesta as well as the presence of eggs in the feces. During the examination of the large intestine contents, most of the *Syphacia* worms found were females, while males were few and seldom seen.

Concerning protozoa, *Cryptosporidium*, *Giardia*, and *Toxoplasma* were diagnosed by rapid a test. In addition, *Cryptosporidium* oocysts were detected in the ZN‐stained fecal smears as small spherical red bodies against the blue background. *Giardia* was not confirmed by another test. Two rats had coinfection with both *Cryptosporidium* and *Giardia*. *Sarcocystis* was detected in the thigh muscles of 13 rats. In the fresh‐squashed muscle preparations, sporocysts were morphologically identical and most likely represented one species. They appeared as spindle‐shaped cysts among the muscle fibers. In the histopathological examination, the cross sections of the sarcocysts were observed with thin walls and septa dividing their chambers into compartments filled with bradyzoites. The species of *Sarcocystis* was determined as *S. cymruensis* by genetic characterization of sarcocysts targeting seven genetic loci; for more details see Aryan et al. [23].

The present study revealed that the overall prevalence of parasitic infection in rodents is influenced by seasonal variations. The infection rate in brown rats was significantly higher (*p* = 0.029) in the wet season than in the dry season; in addition, fewer rats were trapped in the dry season (Table 3).

**TABLE 3:** Overall prevalence of parasitic infection in different seasons in brown rats.

Rodent species	Wet season		Dry season	
	No. exam.	No. infect. (%)	No. exam.	No. infect. (%)
*R. norvegicus*	78	72 (92.3)^a^	20	15 (75%)^b^

*Note:* Different superscript showing significant (*p* < 0.05) differences between seasons in *R. norvegicus*.

Four zoonotic parasite species were detected in brown rats in this study, namely, *R. nana, H. diminuta, T. taeniaeformis,* and *T. gondii*, and 3 parasites (*G. neoplasticum, Cryptosporidium*, and *Giardia*) were considered potentially zoonotic. In addition, seven species were reported for the first time in rodents in Kuwait, namely, *T. crassicuda, G. neoplasticum, Eimeria miyairii, E. nieschulzi, Sarcocystis cymruensis, Cryptosporidium*, and *Giardia*.

## 4. Discussion

Our study and that of Al‐Behbehani [9] showed low diversity of commensal rodents in Kuwait, as only two species, *R. norvegicus* and *M. musculus,* were found in urban areas. In contrast, higher diversity of these rodents was reported in other countries, for example, in Egypt, where El‐Azazy [22] found 7 species, namely, *R. norvegicus*, *Rattus rattus rattus, Rattus rattus alexandrinus, Rattus rattus frugivorus*, *Arvicanthis niloticus*, *Acomys cahirinus*, and *M. musculus*.

Reporting 13 parasites of different species in brown rats in our study could reflect the ecological factors prevailing in the study area with waste accumulation, low levels of hygiene, and improper sewage drainage, leading to a high density of rats, cats, and insects; the situation facilitated the perpetuation and transmission of heteroxenous and monoxenous parasite species. In general, the transmission of parasites in the urban environment seems to be high, as the overall prevalence of parasitic infection was 88.8% and the coinfections of rats with different parasite species reached 55.1%, indicating their susceptibility to repeated infections. As urban settings represent a significant portion of the ecosystem experienced by the human population, parasitic infections carried by brown rats are a candidate for spillover into the human population.

The overall prevalence of parasitic infection was higher in the wet season than in the dry season. This seasonal variation could be related to the relative abundance of brown rats due to climatic conditions in each season. It has been documented that seasonal climatic conditions may exert an influence on rat populations and activity [24]. In the dry season, the high ambient temperatures, which may reach 50°C, limit the activity of brown rats and reduce the availability of infective stages of different parasites and intermediate invertebrate hosts; the situation, which leads to decreasing parasite transmission in the dry season.

*R. nana* and *H diminuta* are common cestodes, and both species are zoonotic. *R. nana* has been reported more frequently in human cases than *H. diminuta* [25] because it can be transmitted directly without needing any intermediate hosts. While *H. diminuta* can only be transmitted to humans by accidental ingestion of infected insects, similarly, in our study brown rats showed a higher infection rate (32.7%) for *R. nana* than for *H. diminuta* (23.5%). In contrast, other studies, for example, [25, 26] reported higher infection rates for *H. diminuta*. Probably, the abundance and high availability of insects to the rodents may lead to a higher frequency of *H. diminuta*. Higher prevalence of *H. diminuta* and *G. neoplasticum* infections in adults compared to young rats may be attributed to the fact that the predatory behavior and skills for catching insects are rarely seen in juvenile rats, but they typically emerge only after puberty [27]. The occurrence of hymenolepidid worms, particularly *R. nana* in brown rats, in this study could pose a threat to humans in urban settlements. *R. nana* was reported in patients attended to at 6 general hospitals in Kuwait City and its suburbs [28].

*C. fasciolaris* is the larval stage of *T. taeniaeformis,* the cestode inhabiting the small intestine of felines. This metacestode has been reported frequently in rodents, including brown rats, in many countries, with prevalence ranging from 4.3% to 67.7% [29]. The relatively high prevalence (23.5%) of *C. fasciolaris* gives an indication that the study area was contaminated with the eggs of *T. taeniaeformis* deposited in the feces of stary cats, which are widespread, roaming in the urban areas of Kuwait [30]. This cestode has zoonotic potential, as humans have been accidentally infected with the adult and metacestode [29].

The pinworm of rats is *S. muris* [31]; however, we could not identify *Syphacia* worms to the species level, as the majority were females, and the accurate identification of this oxyurid must be carried out using the morphometric analysis of male individuals. In the present study, males were rarely detected, probably due to their short life span, only 6 days after fertilization, while females stay longer in the cecum for oviposition [32].

We report *G. neoplasticum* in *R. norvegicus* for the first time in Kuwait. Previous studies found other species of *Gongylonema*, namely *G. minimus* in *M. musculus* and *G. brevispiculum* in *M. libycus*, *M. crassus*, *T. indica*, and *M. musculus* [9].

*Gongylonema* infection has been rarely reported in humans. Most of the human Gongylonemosis cases were due to *Gongylonema pulchrum*, the natural parasite of ruminants. These cases were found mainly in humans living in or visiting rural areas [33]. On the other hand, *G. neoplasticum*, the natural and common parasite of rats, would seem to be much more likely the causative agent of gongylonemosis in urban settlements [34]. Based on the high rates of transmission of *G. neoplasticum* among rats in urban areas in Malaysia [35], Tunisia [36], and Spain [26, 37], it is considered to be potentially zoonotic. In Kuwait, the present report may call the attention of clinicians to consider Gongylonemosis in urban residents showing lesions in the oral cavity or/and a sensation of the movement of a foreign body in the mouth.

In this study *Leishmania* was not detected in brown rats. Leishmaniasis has been reported sporadically in Kuwait, with only 6 papers found in the literature; in contrast, the disease has been reported more frequently in the adjacent countries Saudi Arabia and Iraq [38]. Rodents belonging to the subfamily Gerbillinae are the main reservoirs of *L. major*, while commensal rodents have a minor role in the maintenance of this parasite [39]. In one report from Iran, *L. major* was detected in *R. norvegicus* [40]. In Kuwait, Hussein [41] failed to detect *Leishmania* infection in tissue smears and culturing in rodents.

*Cryptosporidium* was found in only 2% of the brown rats examined. This prevalence is much less than that reported in rats in Spain (13.9%) [42]. Similarly, *Giardia* infection was also reported at low prevalence (2%). Higher infection rates have been found in brown rats in other studies, for example, 28% in Sweden [43]. In the current study, as the study area was crowdedly inhabited by poorly housed migrant workers and provided with mediocre municipal services, probably rats acquired the infection with both parasites in the sewage.

The immunochromatography assay used in this study and other commercial rapid tests were developed to detect the coproantigens of *C. parvum* and *G. intestinalis*, which are the most frequent and pathogenic in humans and livestock. Many studies, for example, Weitzel et at. [44] and Chalmers et al. [45], have shown that the sensitivity rates of these tests vary from 75% to 100% and their specificity may reach above 98%. However, the performance of rapid assays for detection of nonparvum/intestinalis species is less understood [46].

In Kuwait, because of the high prevalence of *C. parvum* among humans and farm animals [47, 48] and the detection of *G. intestinalis* during fecal examination of patients [49], the potential role of brown rats as reservoirs of zoonotic cryptosporidiosis and giardiasis should be considered, and further investigation using molecular tools is recommended.

Only one rat was found to be positive for *T. gondii* using a rapid test. The low prevalence of *Toxoplasma* infection in brown rats could reflect scarcity of *Toxoplasma* infection among cats. Oocysts of this protozoan were detected in the feces of 2.1% of young stray cats in Kuwait [50]. Also, Murata et al. [51] found only one brown rat was infected with *Toxoplasma* in Grenada, and they concluded that rats had little role in the transmission of toxoplasmosis on the island despite its high prevalence among humans and animals.

In Kuwait, little information is available about toxoplasmosis in humans and animals, the topic that needs more investigation. Behbehani and Al‐Karmi [52] reported a high seroprevalence rate (95.5%) of *Toxoplasma* infection among the Kuwaiti population, with higher titers of antibodies in Bedouins compared to urban Kuwaitis and other nationalities. Iqbal and Khalid [53] detected acute *T. gondii* infection in early pregnancy by IgG avidity and PCR analysis. Al‐Karmi and Behbehani [54] detected *Toxoplasma* infection in *Meriones crassus*, the desert rodent, and suggested that these small mammals could be a source of infection to Bedouins.

Limitations of the study, due to the pressure of the work, for example, the examination of all organs of rats for parasites and many of them at the same time, made it impossible to determine the intensity of infections through a quantitative method such as counting worms, eggs, and oocysts. Genotyping of *Cryptosporidium* and *Giardia* spp. could not be done by molecular analysis because of logistical, personnel, and financial reasons.

## 5. Conclusion

Brown rats in Kuwait harbor a wide variety of 13 parasites, with 4 species being zoonotic, 3 species potentially zoonotic, and 7 species reported for the first time, enriching our knowledge of the diversity of rodent parasite fauna and increasing the awareness of the potential risk posed by brown rats to human and animal health. As zoonotic parasites such as *Cryptosporidium, Giardia*, and *Gongylonema* are reported for the first time in rodents in Kuwait, their epidemiology and the role of rats in transmission are topics in need of more investigation. Ecological factors in urban areas shape the brown rat parasite community and facilitate their perpetuation and transmission. Despite the higher overall prevalence of urban rat parasites in the wet season, their risk could be expected to be higher in the dry season due to moving rats to houses of residents seeking shelter from the harsh climatic conditions in this season.

## Author Contributions

Contribution to the conception of the research idea, designing and data collection, formal analysis, interpretation of data, and writing and editing the manuscript: Fatemah A. M. Aryan, Osama Mohammed El‐Shafei El‐Azazy, and Laila Mohammed Azad Tahrani; supervision: Osama Mohammed El‐Shafei El‐Azazy.

## Funding

This research did not receive external funding; the author bore the associated costs.

## Disclosure

All authors have approved the submission of the manuscript.

## Ethics Statement

This work was done according to the regulations set by PAARF for conducting research and received ethical approval from the technical committee of the Animal Resources sector (reference no. 021111, date November 28, 2019, Deputy Director General for Animal Resources). Also, the study received ethical approval from the Ministry of Health to trap and examine rodents for zoonotic parasitic diseases (reference no. 2021‐658‐4, date January 21, 2021, Deputy Minister).

## Conflicts of Interest

The authors declare no conflicts of interest.

## Data Availability

The data that support the findings of this study are available from the corresponding author upon reasonable request.
